# Improved U-Shaped Convolutional Neural Network with Convolutional Block Attention Module and Feature Fusion for Automated Segmentation of Fine Roots in Field Rhizotron Imagery

**DOI:** 10.3390/s25164956

**Published:** 2025-08-11

**Authors:** Yufan Wang, Fuhao Lu, Changfu Huo

**Affiliations:** 1 School of Computer Science and Technology, Tongji University, Shanghai 201804, China; 2252439@tongji.edu.cn; 2 CAS Key Laboratory of Forest Ecology and Management, Institute of Applied Ecology, Chinese Academy of Sciences, Shenyang 110016, China; lufuhao20@mails.ucas.ac.cn; 3 College of Resources and Environment, University of Chinese Academy of Sciences, Beijing 100049, China

**Keywords:** rhizotron image segmentation, attention mechanism (CBAM), U-Net architecture, root phenotyping, transfer learning

## Abstract

Accurate segmentation of fine roots in field rhizotron imagery is essential for high-throughput root system analysis but remains challenging due to limitations of traditional methods. Traditional methods for root quantification (e.g., soil coring, manual counting) are labor-intensive, subjective, and low-throughput. These limitations are exacerbated in in situ rhizotron imaging, where variable field conditions introduce noise and complex soil backgrounds. To address these challenges, this study develops an advanced deep learning framework for automated segmentation. We propose an improved U-shaped Convolutional Neural Network (U-Net) architecture optimized for segmenting larch (*Larix olgensis*) fine roots under heterogeneous field conditions, integrating both in situ rhizotron imagery and open-source multi-species minirhizotron datasets. Our approach integrates (1) a Convolutional Block Attention Module (CBAM) to enhance feature representation for fine-root detection; (2) an additive feature fusion strategy (UpAdd) during decoding to preserve morphological details, particularly in low-contrast regions; and (3) a transfer learning protocol to enable robust cross-species generalization. Our model achieves state-of-the-art performance with a mean intersection over union (mIoU) of 70.18%, mean Recall of 86.72%, and mean Precision of 75.89%—significantly outperforming PSPNet, SegNet, and DeepLabV3+ by 13.61%, 13.96%, and 13.27% in mIoU, respectively. Transfer learning further elevates root-specific metrics, yielding absolute gains of +0.47% IoU, +0.59% Precision, and +0.35% F1-score. The improved U-Net segmentation demonstrated strong agreement with the manual method for quantifying fine-root length, particularly for third-order roots, though optimization of lower-order root identification is required to enhance overall accuracy. This work provides a scalable approach for advancing automated root phenotyping and belowground ecological research.

## 1. Introduction

Fine roots (diameter < 2 mm) are the primary organs for water and nutrient uptake in plants, whose dynamics directly influence carbon allocation and productivity in forest ecosystems [1]. As a keystone species, the response of *Larix* spp. fine roots to climatic factors are particularly crucial for understanding boreal forest carbon cycling across the Northern Hemisphere [2]. Traditional fine-root-monitoring methods (e.g., soil coring or excavation, manual counting) suffer from high destructiveness, low data throughput, and substantial subjective errors [3], limiting their applicability for long-term in situ observations. Recent advances in rhizotron and minirhizotron technologies have enabled non-destructive imaging for fine-root phenotyping [4]. However, automated segmentation of fine roots, particularly the target *Larix* species in this study, within field-acquired rhizotron imagery remains a significant challenge. Complex soil environments, uneven illumination, and dynamic morphological changes of fine roots in time-series imagery pose difficulties for accurate automated segmentation and quantification [5]. Crucially, the segmentation task primarily relies on distinguishing subtle visual attributes of fine roots (e.g., texture patterns, branch structures, and intensity contrast relative to the heterogeneous soil background), which are often obscured by noise and occlusion.

Traditional image processing techniques (e.g., threshold segmentation, edge detection) are frequently prone to false positives and misses due to soil particle interference [6]. For instance, the local entropy threshold method proposed by Zeng et al. [7] exhibits limited robustness for low-contrast fine-root segmentation due to blurred boundaries between fine roots and humus. The rapid development of deep learning has recently revolutionized semantic segmentation for root images. The U-Net, featuring encoder–decoder architectures with skip connections [8], has shown exceptional performance in agricultural imaging [9,10]. Xu et al. [11] enhanced soybean root identification by incorporating a multi-scale feature pyramid module based on U-Net architecture. Wang et al. [12] used SegRoot—a SegNet-based framework enabling high-throughput in situ soybean root analysis from minirhizotron images. Despite these achievements, existing studies have predominantly focused on static single-timepoint segmentation, lacking robust modeling of temporal dynamics [13]. This limitation is especially pronounced when dealing with field-acquired in situ rhizotron images of *Larix* fine roots, characterized by intricate root morphology, blurred root–soil boundaries and frequent omission of fine root branches during segmentation.

Research on automated segmentation of *Larix* fine roots in temporal rhizotron imagery faces two primary challenges: (1) fine roots exhibit significant morphological variations across inter-annual fluctuation and seasonal patterns [2], demanding robust multi-scale feature extraction capabilities [14]; (2) heterogeneity between time-series root images may lead to the failure of traditional data augmentation strategies, and transfer learning and cross-domain adaptation techniques can effectively alleviate such problems [15]. Although Smith et al. [16] enhanced model generalizability through a fine-grained annotation refinement, their approach inadequately addresses spatiotemporal correlations in time-series data. While Dadi et al. [17] improved model robustness through synthetic data, synergistic strategies combining mixed-species training with transfer learning require further exploration [18]. Resolving these limitations is critical for advancing dynamic root monitoring systems and providing novel methods for carbon flux modeling in boreal forests.

To our knowledge, while deep learning models exist for root segmentation, none integrate CBAM for field-acquired time-series rhizotron images with heterogeneous soil backgrounds. Therefore, the objectives of this study focus on developing a robust segmentation method specifically for *Larix* fine roots in in situ field rhizotron imagery. We hypothesize that integrating CBAM for channel–spatial attention and UpAdd for feature fusion will significantly improve segmentation accuracy for fine roots in noisy field rhizotron imagery, particularly for low-contrast regions and blurred boundaries. Our core research question is how deep learning architectures can be optimized to effectively segment fine roots based on their complex visual attributes (e.g., texture, contrast, morphology) within noisy, heterogeneous field soil backgrounds, while leveraging cross-species knowledge. To address this, we propose a method grounded in architectural refinements through (1) developing a multi-scale U-Net integrated with attention mechanisms (CBAM) to enhance focus on discriminative root features (edges, texture, low-contrast branches) against a complex background; and (2) leveraging publicly available multi-crop datasets (e.g., PRMI [19]) for model pre-training to boost generalizability across plant species by transfer learning fusion. This work establishes a novel approach for deep learning applications in multi-source temporal root data analysis, aiming to contribute significantly to more precise forest carbon cycle modeling and the development of high-throughput root phenotyping platforms for ecological research. The practical interest lies in providing a scalable, automated tool for quantifying fine root dynamics in field studies, overcoming the limitations of manual methods.

## 2. Materials and Methods

### 2.1. Image Acquisition and Annotation

This study employs two root image datasets, a single-species dataset and a mixed-species dataset. The basic characteristics of these datasets are summarized in Table 1.

Single-species dataset: In situ fine root images of larch (*Larix gmelinii*) were collected from glass rhizotrons installed in a 50-year plantation at the Qingyuan Forest Ecosystem National Observation and Research Station (41°51′9.94″ N, 124°56′11.22″ E) in Liaoning Province, China (Figure S1). The imaging system consisted of a rhizotron–digital scanning setup, including glass rhizotrons (20 cm × 30 cm, width × depth), a flat scanner, battery power, and a laptop computer [20]. Glass rhizotrons were installed in October 2011, and root images (24-bit color, 600 dpi) were sampled from May 2012 and August 2017. These images represent a time series of root development, with variations in root morphology captured at observation periods (Table S1). A total of 104 root images were manually processed using Rootfly software (v2.0.2, GPL, Clemson University, Clemson, SC, USA) to extract trait values of fine root length. Of these, 82 images with high signal-to-noise ratios were selected for manual annotation. Annotations were performed using Adobe Photoshop CC 2020 (Adobe Inc., San Jose, CA, USA), where fine roots were labeled by filling them in white, and the soil background was masked in black. The annotation time for each image was approximately 3 h. The resolution of all annotated images was further standardized to 1275 × 1755 pixels. All annotated images were then segmented into smaller 512 × 512-pixel patches for training using a sliding window approach with a stride of 256 pixels. Areas at the edges insufficient to form a full patch were filled with black padding to maintain consistent dimensions. After cropping, a total of 1968 small image patches were obtained. These patches were randomly divided into training, validation, and test subsets in a 7:2:1 ratio, resulting in 1377, 393, and 198 images for each respective set.

Mixed-species dataset: This dataset originates from the minirhizotron dataset presented in the PRMI paper [19], accessible at “https://gatorsense.github.io/PRMI/” (accessed on 21 April 2025). The original PRMI dataset comprises minirhizotron images of six crop species, including cotton, papaya, and sunflower, captured across multiple time points and soil depths. It contains over 63,000 manually annotated images. For this study, images of five crops—cotton, peanut, sesame, papaya, and sunflower—were selected.

### 2.2. Image Preprocessing and Data Augmentation

The original images and the corresponding annotated images were read from the input data list and loaded using OpenCV. Both the original and annotated images were uniformly resized to 256 × 256 pixels to ensure dataset consistency. During image preprocessing, color information was transformed from RGB to HSV space to enhance root–soil contrast. Pixel values were normalized to the [0, 1] range, and we adjusted the image dimension order to match PyTorch’s input format, i.e., transforming from (H, W, C) to (C, H, W). For annotated images, preprocessing involves (1) converting color label maps to grayscale; (2) performing normalization to distribute pixel values within [0, 1]; (3) applying threshold-based binarization to convert pixels to 0 or 1; and (4) adding a channel dimension to reshape the tensor to (1, H, W), meeting model input requirements.

To improve model generalization for larch roots under diverse field conditions, we developed a specialized augmentation strategy based on our time-series rhizotron image dataset, which exhibited significant variations in root morphology and soil backgrounds. Following the method of Tang et al. [13], four augmentations were implemented: (1) randomly altering brightness, contrast, and saturation up to ±50% to enhance the robustness against color variations; (2) randomly cropping (scales between 0.2 and 1.0) followed by resizing to 256 × 256 pixels to enhance the model’s identification capacity in certain areas; (3) rotating images by random angles within ±90° to increase adaptability to root orientation changes; and (4) horizontally or vertically flipping images with a fixed probability to improve generalization for mirrored variations.

During the training phase, the data loading function shuffles the samples randomly and independently applies each of the aforementioned augmentation methods with a probability of 0.3. If an augmentation method is selected, its corresponding transformation is applied to the image. As these augmentation strategies are mutually independent, multiple transformations may be applied simultaneously in a single data loading step. This substantially enhances the diversity of the training samples and the model’s robustness against variations in input data.

### 2.3. Proposed Larch Root Image Segmentation Model

#### 2.3.1. Structure of Improved U-Net Architecture

Building upon original U-Net architecture [8], this study introduces an improved network designed to enhance feature expression capabilities. The core modifications are (1) the integration of a Convolutional Block Attention Module (CBAM) for adaptive weighting of feature channels and spatial locations [21], and (2) the embedding of a novel feature extraction and fusion module (UpAdd) within the skip connections between the encoder and decoder [22]. The encoder consists of four stages with output channels of 64, 128, 256, and 512, respectively, followed by a 1024-channel bottleneck. The decoder mirrors the encoder, with skip-connections fused via UpAdd modules.

**Figure 1:**
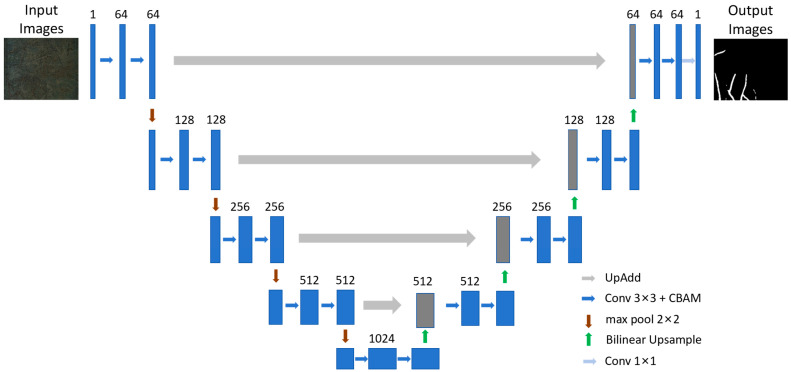
Overall structure diagram of root image segmentation model.

The improved U-Net retains the symmetric encoder–decoder structure (Figure 1). The encoder consists of four stacked DoubleConv blocks. Each DoubleConv block performs two successive 3 × 3 convolution operations, each followed by Batch Normalization (BN) and a ReLU activation function. These sequential convolutions extract increasingly discriminative features. The resulting features are then processed by a CBAM module, which applies attention weighting to enhance the model’s focus on critical channels and spatial regions. After each DoubleConv + CBAM stage, a 2 × 2 max pooling operation downsamples the feature maps, halving their spatial dimensions while doubling the number of channels to increase semantic capacity.

**Figure 2:**
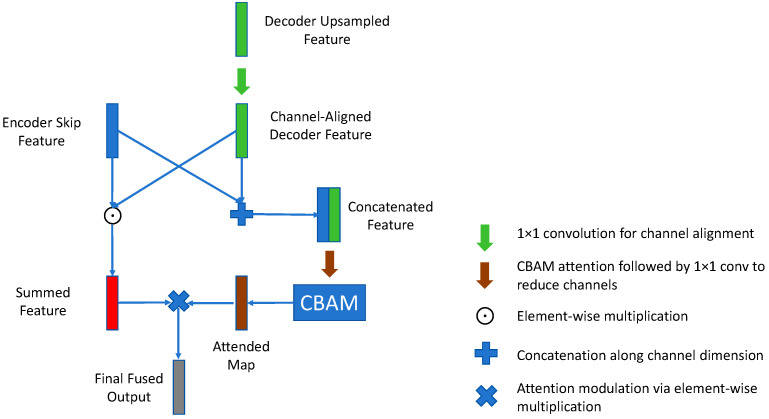
Specific process of upsampling.

The decoder progressively restores spatial resolution through upsampling and incorporates corresponding feature information from the encoder via skip connections. While the original U-Net utilizes direct concatenation for feature fusion, this approach can introduce feature redundancy and semantic misalignment. To address this limitation, we designed the UpAdd module (detailed in Figure 2), which operates as follows: (1) applying bilinear interpolation upsampling to the output from the previous decoder layer; (2) using a 1 × 1 convolution to adjust the channel dimension of the upsampled features (${\hat{E}}^{\left(l\right)}$) to match that of the corresponding encoder skip connection feature ($S^{\left(l\right)}$), ensuring channel alignment; (3) fusing $S^{\left(l\right)}$ and ${\hat{E}}^{\left(l\right)}$ via element-wise addition ${\hat{E}}^{\left(l\right)}+S^{\left(l\right)}$; (4) concatenating ${\hat{E}}^{\left(l\right)}$ and $S^{\left(l\right)}$ and feeding the result into the CBAM module to generate a joint channel–spatial attention weight map $CBAM(Concat(S^{\left(l\right)},{\hat{E}}^{\left(l\right)})$); (5) modulating the element-wise summation result from step 3 using $CBAM(Concat(S^{\left(l\right)},{\hat{E}}^{\left(l\right)})$) via element-wise multiplication (see Equation (1)); and (6) processing the weighted features through a DoubleConv block to further refine high-quality fused features.

(1) $$U^{\left(l\right)}=ConvBlock((S^{\left(l\right)}+{\hat{E}}^{\left(l\right)})⊙CBAM(Concat(S^{\left(l\right)},{\hat{E}}^{\left(l\right)})))$$

(2) $${\hat{E}}^{\left(l\right)}=Align\left(Upsample(U^{\left(l+1\right)})\right)$$

where $S^{\left(l\right)}$: features from the encoder skip connection; ${\hat{E}}^{\left(l\right)}$: decoder feature after upsampling and channel alignment; ⊙: element-wise multiplication; upsample: bilinear interpolation upsampling operation; Align: the 1 × 1 convolution operation for channel alignment.

#### 2.3.2. Convolutional Block Attention Module (CBAM)

We employed the original Convolutional Block Attention Module (CBAM) proposed by Woo et al. [23], which sequentially applies channel and spatial attention to adaptively refine intermediate features. In our network, CBAM was applied after both convolutional and activation operations within each DoubleConv block, allowing the attention mechanism to operate on high-level feature representations, as well as in the UpAdd fusion module, enabling the model to focus on the most informative feature regions and channels. CBAM is a lightweight and efficient attention mechanism employing sequential channel attention and spatial attention submodules (Equation (3)).

Channel attention: This submodule evaluates the importance of each feature channel. For an input feature map (*F*), it generates two channel descriptors by global average pooling and global max pooling. Both descriptors are processed by a shared-weight Multi-Layer Perceptron (*MLP*). The *MLP* outputs are element-wise additions, and a sigmoid activation function generates the channel attention weight vector (*Mc*) (Equation (4)), used to recalibrate channel responses.

Spatial attention: Operating on the channel-weighted feature map (*F*′), this submodule identifies spatially significant regions. It computes average-pooled and max-pooled features along the channel dimension, yielding two single-channel (H × W) maps. These maps are concatenated channel-wise and processed by a 7 × 7 convolution layer to generate the spatial attention weight map (*Ms*) (Equation (5)). This map modulates the feature map, directing the network’s focus to key spatial locations. Regarding how to integrate CBAM, channel attention, and spatial attention into U-net, please refer to Supplementary Materials for the code implementation (Table S2).

(3) $$CBAM=M_{s}(M_{c}(F)\cdot F)$$

(4) $$M_{c}(F)=\sigma (MLP(AvgPool(F))+MLP(MaxPool(F)))$$

(5) $$M_{s}(F^{\prime })=\sigma (f^{7\times 7}([AvgPool(F^{\prime });MaxPool(F^{\prime })]))$$

where σ is the sigmoid function, F in the input feature map, MLP is a two-layer fully connected perception, $F^{\prime }$ is the channel-weighted feature map, and $f^{7\times 7}$ denotes a 7 × 7 convolution operation.

### 2.4. Model Training

#### 2.4.1. Experimental Environment

All experiments were conducted on an Ubuntu 20.04 LTS system equipped with an NVIDIA GeForce RTX 4090 graphics card (sourced from the AutoDL platform, a cloud service provider based in Nanjing, Jiangsu, China). It was also equipped with CUDA 11.8 as the parallel computing framework and CUDNN 8.7.0 as the deep neural network acceleration library. The implementation was based on Python 3.8.10 and PyTorch 2.0.0, with the following software configurations and packages: Opencv-Python 4.7.0.72, NumPy 1.24.4, Pillow 9.4.0, scikit-image 0.21.0, and scikit-learn: 1.3.2.

All models were trained using a batch size of 16 and an input image resolution of 512 × 512 pixels. The optimizer used was AdamW, configured with momentum parameters (β_1_ = 0.9, β_2_ = 0.999), a learning rate of 0.0001, and a weight decay of 0.0001. A cosine annealing learning rate scheduler (T_max = 80) was applied across 80 training epochs to gradually decay the learning rate.

#### 2.4.2. Loss Function

To enhance the segmentation performance for the fine-grained structure in complex backgrounds, we designed a hybrid loss function termed BCEDiceFocalEdgeLoss (*L*_total_), which addresses three critical segmentation challenges: class imbalance, boundary ambiguity, and structural fragmentation. Specifically, the loss function integrates three complementary components: (1) Dice Loss—optimizing overlap between predictions and ground truth, particularly effective for small foreground objects [24]; (2) Focal Loss—prioritizing hard-to-classify samples (e.g., transitional root-edge regions) while suppressing well-classified background pixels, with parameter (α = 0.9, γ = 2.5)-emphasizing hard regions [25]; and (3) Edge Loss—reinforcing boundary learning by assigning higher weights to edge regions identified via Sobel filtering on the ground truth masks, improving performance on blurred or fragmented root edges [26]. The final loss is formulated as the weighted sum of the three components, as shown in Equation (6).

(6) $$L_{total}=w_{1}\cdot L_{Focal}+w_{2}\cdot L_{Dice}+w_{3}\cdot L_{Edge}$$

where *w*_1_, *w*_2_, and *w*_3_ are 0.4, 0.4, and 0.2, respectively.

### 2.5. Experimental Strategy

To systematically evaluate the performance and generalization capability across data distributions, this study designs three experimental strategies: an ablation study, a comparative experiment, and a transfer learning experiment. This approach provides both quantitative performance metrics and qualitative insights into the model’s architectural advantages. The single-species dataset was used in ablation studies and comparative experiments, while the mix-species dataset (PRMI) was only used in transfer learning experiments.

#### 2.5.1. Ablation Study

To address information loss during downsampling and limited identification of fine root structures in original U-Net, configurations of ablation studies were conducted focusing on two critical components: feature extraction and feature fusion. The configurations of the CBAM attention mechanism, the SE (Squeeze-and-Excitation) attention mechanism, or the standard convolution (Conv) were compared for feature extraction. Two strategies of channel concatenation (Concat) or feature addition (Add) were evaluated for feature fusion. All ablations were performed on a single dataset, employing data augmentation during training and initializing model parameters randomly. As detailed in Table 2, the configuration of CBAM + Conv2 + Add achieved optimal segmentation performance.

#### 2.5.2. Comparative Experiment

We compared our improved U-net against three state-of-the-art semantic segmentation models—SegNet, DeeplabV3Plus, and PSPNet. All models were trained under identical hyperparameters (including 80 epochs) and evaluated on consistent test sets, with particular emphasis on segmentation accuracy for challenging fine-grained root structures.

#### 2.5.3. Transfer Learning Experiment

Transfer learning is employed to enhance model performance in scenarios with limited training data, enhancing identification ability, and generalization under varied root morphologies and complex backgrounds. Here, we used the opening PRMI dataset for transfer learning to enhance generalization across species and soil conditions, mitigating overfitting to larch-specific features. We compared plain training with transfer learning experiments under two schemes: (1) models (original U-Net and improved U-Net) were trained directly on mixed datasets without pretrained weights and (2) the improved U-Net was pretrained on a single dataset and fine-tuned pretrained weights on mixed datasets. Mixed datasets comprised minirhizotron images from multiple crops (Table 1) to enhance structural and background generalization.

For all validation phases, the predicted probability maps were binarized by thresholding, and we evaluated a range of thresholds from 0.1 to 0.9 (step = 0.1). The final performance metrics were reported based on the threshold that achieved the highest mean intersection over union (mIoU) across the validation set, ensuring a fair and robust comparison among different models and settings.

### 2.6. Model Performance Evaluation

This study employs four standard metrics to evaluate the model’s performance: Precision (*P*), Recall (*R*), intersection over union (*IoU*), and F1-Score (*F*1). Their calculation formulas are as follows:

(7) $$Precision=\frac{TP}{TP+FP}$$

(8) $$Recall=\frac{TP}{TP+FN}$$

(9) $$IoU=\frac{TP}{TP+FN+FP}$$

(10) $$F1=\frac{2\times Precision\times Recall}{Precision+Recall}$$

where *TP*: True Positive, denoting root pixels correctly predicted as roots; *FP*: False Positive, denoting background pixels misclassified as roots; and *FN*: False Negative, denoting root pixels misclassified as background.

All metrics range between 0 and 1, with higher values indicating superior performance. The intersection over union (*IoU*) evaluates spatial overlap—the similarity between the predicted pixel classification and the ground truth. Precision and Recall measure classification correctness and completeness, and the *F*1-score provides a balanced performance summary—the harmonic mean of Precision and Recall.

### 2.7. Statistical Analysis

To evaluate the agreement between manual line–intersect measurements and improved U-Net segmentation in root length quantification, Pearson’s correlation coefficient (r) was calculated separately for first-order, second-order, and third-order roots. This stratification by root order allowed for assessment of segmentation accuracy across different root orders. Statistical significance was determined at α = 0.05, with Bonferroni correction applied to account for multiple comparisons.

## 3. Results

### 3.1. Segmentation Performance of Improved U-Net in Ablation Study

The improved U-Net model (Conv2 + CBAM + Add) achieved optimal performance across comprehensive evaluation metrics (Table 3). Compared to the original U-Net, it exhibited significant improvements of 12.04%, 5.03%, 35.28%, and 11.85% in mIoU, mRecall, root F1-score, and mPrecision, respectively. Notably, the proposed model outperformed all variants in two critical root-specific metrics—the highest scores in Root IoU (42.66%) and Root Recall (75.18%). Compared with CBAM, SE exhibited severe limitations under the Add operation. SE is lower in key parameters of R F1, R IoU, and R Precision. Especially, R Recall is 9.54% lower than CBAM (Table 3), indicating that SE has a higher missed detection rate for fine roots. These results demonstrate its superior ability in identifying and localizing fine root regions within complex backgrounds.

### 3.2. Segmentation Performance on Different Methods in Comparative Experiment

The improved U-Net surpassed mainstream semantic segmentation models (PSPNet, SegNet, DeepLabV3+) in all key metrics (Table 4). Its mIoU (70.18%), mRecall (86.12%), and mPrecision (75.89%) outperformed those of its counterparts. Specifically, the F1-score (58.71%) of our proposed model exceeded those of PSPNet, SegNet, and DeepLabV3+ by 1.32 times, 1.45 times, and 1.26 times, respectively (Table 4).

**Figure 3:**
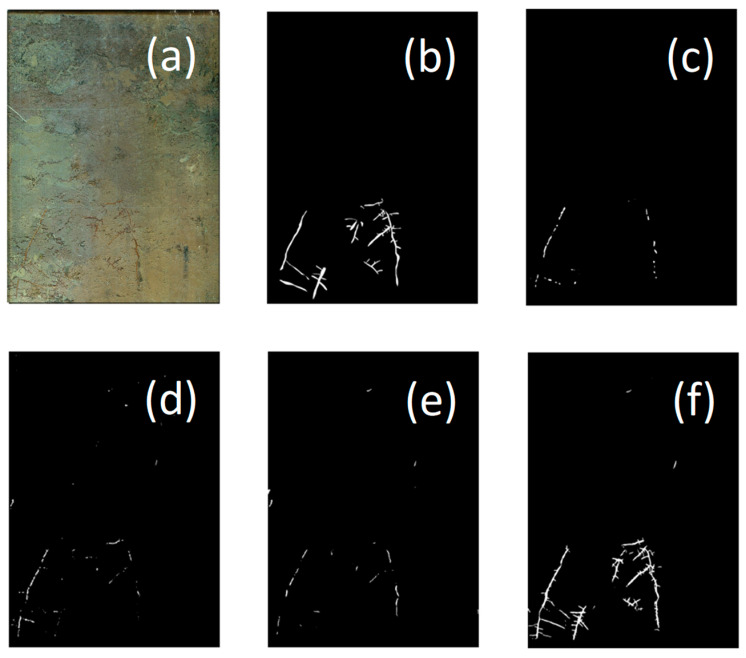
Comparison of segmentation performance with different methods. (**a**) Original image; (**b**) Annotated image; (**c**) PSPNet; (**d**) SegNet; (**e**) DeepLabV3+; (**f**) Improved U-net.

These quantitative findings are further supported by visual comparisons in Figure 3. The improved U-Net completely reconstructed contours of larch fine roots and successfully segmented most of the fine root branches (Figure 3f). In contrast, the competing models of PSPNet, SegNet, and DeepLabV3+ suffered from severe omission errors and root fragmentation (Figure 3c–e), particularly in regions with blurred edges or weak textures.

### 3.3. Transfer Learning Validation

**Figure 4:**
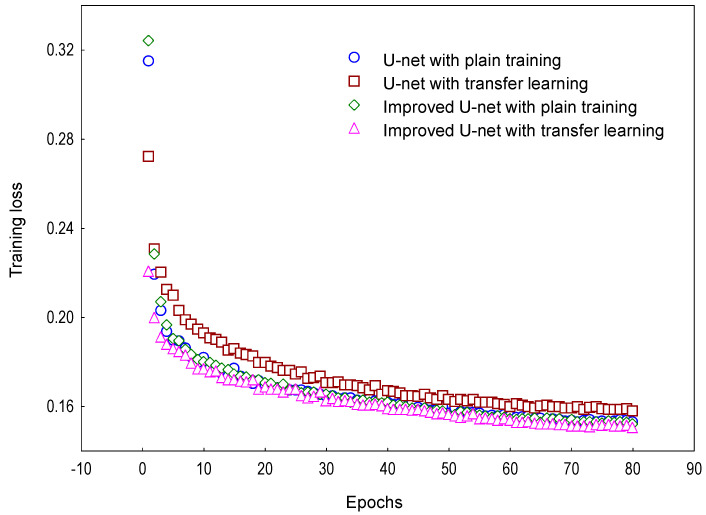
The variation of training loss in plain training and transfer learning.

Figure 4 shows distinct loss evolution patterns between plain training and transfer learning throughout the training process. The improved U-Net model employing transfer learning achieved the lowest overall loss and fastest convergence, validating its superior parameter initialization via pre-trained weights and enhanced fitting efficacy.

As shown in Table 5, the improved U-Net achieved optimal performance under transfer learning. Compared to plain training, it exhibited improvements of 0.18% in mIoU, 0.24% in mPrecision, and 0.35% in Root F1-score, demonstrating enhanced robustness and generalization. When benchmarked against the original U-Net, the improved model delivered gains of 1.21% in mIoU, 0.79% in mPrecision, and 2.16% in Root F1-score, with particularly stable performance at fine-root boundaries. Notably, root-specific identification metrics showed more significant advances: +3.06% in Root IoU, +1.48% in Root Recall, and +1.83% in Root Precision, confirming its heightened accuracy for root segmentation in complex backgrounds.

### 3.4. Fine Root Length

Table 6 compares fine-root length measurements from 10 test images between the manual line–intersect and the improved U-Net segmentation. That total root length exhibited a moderate positive correlation (r = 0.715, *p* = 0.020; r^2^ = 0.512), indicating good overall agreement between U-Net segmentation and the manual method, though only ~51% of variance was explained. There was a strong positive correlation for third-order roots (r = 0.880, *p* < 0.001), but a non-significant correlation for first-order roots (r = 0.270, *p* = 0.450) and second-order roots (r = 0.106, *p* = 0.771). Collectively, the improved U-Net provided excellent measurements for third-order roots but showed the least reliability on first-order and second-order roots.

## 4. Discussion

### 4.1. Enhanced Segmentation Performance Through Architectural Optimization

In situ minirhizotron/rhizotron technology serves as a core method for dynamically quantifying root ecological processes [27,28,29]. However, the substantial volume of time-series images generated by this technique is often compromised by noise and complex soil backgrounds, presenting a critical bottleneck for high-throughput, precise root segmentation [14,30]. For example, Larch fine roots exhibit complex branching structures, where growth dynamics of roots ranging from the first to fifth order can be observed simultaneously within a single rhizotron image [2,20]. This complexity, combined with the highly variable field conditions, significantly impacts segmentation model accuracy. This study addresses the challenge of processing low-quality images by proposing an improved U-Net segmentation model, integrated with transfer learning, specifically for fine root images of *Larix* species acquired using field-based rhizotrons.

To validate the proposed model’s performance, we conducted systematic evaluations on the dataset of larch fine-root images collected through in situ rhizotron time-series observations. Ablation studies revealed that incorporating attention mechanisms (CBAM, SE) significantly improved the Intersection over Union (IoU) and F1 scores for root regions compared to the original U-Net (Conv2 + Concat), confirming their effectiveness in enhancing the recognition of fine-grained root structures (Table 3). SE exhibited severe limitations under the Add operation for root-specific metrics, highlighting that CBAM’s dual channel–spatial attention outperformed SE’s channel-only focus [18], likely by suppressing soil texture noise while enhancing critical recall and root edges (Figure 3f). Furthermore, replacing the Concat feature fusion strategy with UpAdd operations yielded notable improvements in key metrics (e.g., R Precision, mIoU), underscoring the importance of optimized fusion strategies. Consequently, the final improved model architecture (CBAM + Conv2 + UpAdd) demonstrated superior segmentation performance under conditions of complex backgrounds and blurred root boundaries (Table 3).

Comparative experiments further substantiated the superiority of the improved U-Net model, showing consistently better segmentation results than those of mainstream models like PSPNet, SegNet, and DeepLabV3+ (Table 4). These performance differences stem from inherent architectural characteristics: PSPNet relies on Pyramid Pooling Modules (PPM) to capture large-scale contextual information [31] but exhibits insufficient sensitivity to fine-scale structures like fine roots. DeepLabV3+ utilizes atrous convolutions to expand the receptive field; however, its backbone network prioritizes high-level semantic feature extraction [32], often leading to the loss of fine root edge details. SegNet employs a relatively simpler structure, rebuilding features via pooling indices [33], but lacks effective mechanisms to suppress background noise and focuses on critical root regions (Figure 3c–e). In contrast, our improved U-Net demonstrated significant advantages in fine-root Recall rate (R Recall) and overall segmentation accuracy (Figure 3f), providing a more reliable basis for studying in situ fine-root dynamics at the stand and ecosystem scale.

### 4.2. Model Generalization Boost via Transfer Learning

The transfer learning strategy integrated into this study effectively enhanced the model’s generalization capability. As shown in Table 5, applying transfer learning to a mixed-species dataset significantly boosted the root identification performance (R IoU, R Recall, R Precision) of the improved U-Net. This finding is consistent with the studies of Yu et al. [15] and Tang et al. [13], demonstrating that leveraging pre-trained weights for transfer learning robustly improves model adaptability and segmentation performance on complex and diverse root imagery. The improved model surpassed the original U-Net across all core metrics (mIoU, mPrecision, R F1), particularly excelling at distinguishing boundaries between primary and fine roots. This validates the efficacy of combining architectural enhancements with transfer learning.

### 4.3. Limitations and Perspectives

Despite the progress achieved, limitations persist with the proposed improved U-Net model for segmenting larch fine roots. Segmentation performance degrades for lower-order roots (e.g., first- and second-order roots) (Table 6), particularly under conditions of low-contrast, field-based time-series observations via in situ rhizotron. This limitation primarily arises from two factors: (1) insufficient representation—the training datasets contain a disproportionately small number of annotated samples featuring low-contrast, lower-order fine roots; and (2) training bias—the model training predominantly focuses on clearly discernible images of higher-order fine roots (e.g., third-order roots).

Future research could focus on the following areas for improvement: (1) explore more powerful feature extractors or design specialized attention/fusion modules (e.g., Temporal Attention mechanism) to enhance model capacity for extracting fine-scale features of lower-order fine roots; (2) expand the dataset with low-contrast fine-root samples accompanied by fine-grained annotations [16]; and (3) integrate multi-source data (e.g., hyperspectral information or temporal features) [34,35] or employ Generative Adversarial Networks (GANs) and YoloV8n-seg for data augmentation [15,36] to overcome the limitations of a single, relatively small dataset. These efforts should collectively enhance model generalization, segmentation accuracy, and reliability in complex field environments.

## 5. Conclusions

To address the challenge of traditional root image extraction relying on manual annotation—a time-consuming and inefficient process with low-quality images—this study employs deep learning to achieve efficient, automated segmentation of larch fine roots using in situ time-series rhizotron images and open-source minirhizotron multi-crop datasets. The key findings show that an improved U-Net model (Conv2 + CBAM + UpAdd) integrating CBAM attention and additive feature fusion was developed. Ablation studies confirmed that the comprehensive metrics (mIoU: 70.18%; mRecall: 86.72%; mPrecision: 75.89%) surpassed those of the original U-Net, with root-specific IoU and Recall outperforming all variants. Comparative experiments demonstrated that the improved U-Net exceeds PSPNet, SegNet, and DeepLabV3+ across core metrics. The F1-score (58.71%) of our proposed model was higher than those of PSPNet, SegNet, and DeepLabV3+ by 1.32 times, 1.45 times, and 1.26 times, respectively. Furthermore, transfer learning further elevated performance, increasing R IoU, R Precision, and R F1 by 0.47%, 0.59%, and 0.35%, respectively. Notably, root-specific identification metrics (R IoU, R Recall and R Precision) increased by 3.06%, 1.48%, and 1.83%, confirming adaptability and robustness to multi-source root imagery. The improved U-Net showed strong agreement with manual quantification for fine-root length, particularly for higher-order roots (e.g., third-order), though the identification of lower-order roots requires refinement to enhance overall accuracy.

To summarize, the proposed U-Net architecture excels at segmenting larch fine roots, particularly in preserving edges and identifying weak features within in situ rhizotron images. Current limitations include insufficient diversity in training samples and suboptimal segmentation of lower-order fine roots. Future work should focus on: (1) expanding dataset scale and diversity to include multi-species and multi-soil environments; and (2) incorporating multi-source spectral data and refining network architecture to enhance feature identification capacity in segmenting fine-root regions against complex backgrounds.

## Figures and Tables

**Table 1:** Basic characteristics of rhizotron images in the single-species dataset and minirhizotron images in the mixed-species dataset.

Data Category	Single-Species Dataset	Mixed-Species Dataset				
	Larch	Cotton	Peanut	Sesame	Papaya	Sunflower
Resolution/pixel	512 × 512	736 × 552	736 × 552	640 × 480	640 × 480	640 × 480
Training set/sample number	1377	1271	282	10,087	1438	2211
Validation set/sample number	393	564	131	3413	318	722
Test set/sample number	198	577	133	3542	404	967

**Table 2:** Configuration and description for ablation study.

Configuration	Description
Conv2 + Concat	Original U-Net: Dual convolutions followed by encoder–decoder concatenation
Conv2 + UpAdd	Dual convolutions + element-wise addition of encoder–decoder features
SE + Conv2 + Concat	SE attention after dual convolutions + concatenation
SE + Conv2 + UpAdd	SE attention after dual convolutions and concatenation + element-wise addition + concatenation
CBAM + Conv2 + Concat	CBAM attention after dual convolutions + concatenation
CBAM + Conv2 + UpAdd	CBAM attention after dual convolutions and concatenation + element-wise addition + concatenation

**Table 3:** Evaluation metrices of each configuration in ablation study (R: root class; B: background class; m: mean value across all classes; IoU: intersection over union; Recall: sensitivity; Precision: positive predictive value).

Metrics	Conv2 + Concat (Original U-Net)	Conv2 + SE + Concat	Conv2 + CBAM + Concat	Conv2 + UpAdd	Conv2 + SE + UpAdd	Conv2 + CBAM + UpAdd (Improved U-Net)
R IoU/%	28.85	41.33	40.05	42.08	37.23	42.66
B IoU/%	96.43	97.59	97.62	97.65	97.92	97.70
mIoU/%	62.64	69.46	68.84	69.87	67.57	70.18
R Recall/%	67.95	74.23	73.63	73.66	65.64	75.18
B Recall/%	97.20	98.11	98.18	98.26	98.43	98.24
mRecall/%	82.57	86.17	85.90	85.96	82.03	86.72
R Precision/%	36.53	50.65	49.58	52.85	45.68	52.35
B Precision/%	99.18	99.46	99.42	99.36	99.47	99.43
mPrecision/%	67.85	75.05	74.50	76.11	72.58	75.89
R F1/%	43.40	57.33	56.05	58.04	51.25	58.71
B F1/%	98.16	98.77	98.79	98.80	98.94	98.83

**Table 4:** Comparison of segmentation performance on different models (R: root class; B: background class; m: mean value across all classes; IoU: intersection over union; Recall: sensitivity; Precision: positive predictive value).

Metrics	Conv2 + CBAM + Add	PSPNet	SegNet	DeepLabV3+
R IoU/%	42.66	15.83	14.76	16.10
B IoU/%	97.70	97.30	97.68	97.73
mIoU/%	70.18	56.57	56.22	56.91
R Recall/%	75.18	19.78	17.70	18.44
B Recall/%	98.24	99.62	99.77	99.89
mRecall/%	86.72	59.70	58.73	59.16
R Precision/%	52.35	60.88	57.00	71.88
B Precision/%	99.43	97.66	97.91	97.84
mPrecision/%	75.89	79.27	77.45	84.86
R F1/%	58.71	25.28	23.94	25.91
B F1/%	98.83	98.62	98.82	98.84

**Table 5:** Evaluations of plain training and transfer learning in mixed-species datasets (R: root class; B: background class; m: mean value across all classes; IoU: intersection over union; Recall: sensitivity; Precision: positive predictive value).

Metrics	U-Net Plain Training	U-Net Transfer Learning	Improved U-NetPlain Training	Improved U-Net Transfer Learning
R IoU/%	59.92	58.50	60.01	60.29
B IoU/%	97.99	97.90	98.00	98.01
mIoU/%	78.95	78.20	79.01	79.15
R Recall/%	79.29	77.76	79.23	78.91
B Recall/%	98.63	98.58	98.64	98.65
mRecall/%	88.96	88.17	88.94	88.78
R Precision/%	68.76	68.28	69.12	69.53
B Precision/%	99.32	99.28	99.33	99.33
mPrecision/%	84.04	83.77	84.23	84.43
R F1/%	71.97	70.74	72.02	72.27
B F1/%	98.97	98.92	98.98	98.98

**Table 6:** Fine root length from the improved U-Net segmentations and manual line–intersect counts for the 10 test images. Total_length: total root length; Order_1: first-order root; Order_2: second-order root; Order_3: third-order root.

Image	Manual Line–Intersect (mm)				Improved U-Net Segmentation (px)			
	Order_1	Order_2	Order_3	Total_Length	Order_1	Order_2	Order_3	Total_Length
1	603	454	186	1242	1780	380	2345	4505
2	401	395	186	1981	618	149	1390	2157
3	227	276	198	1701	1176	357	2956	4489
4	290	271	303	1864	1849	677	2735	5261
5	266	295	303	2064	2454	922	2825	6201
6	181	238	303	2722	1516	501	2982	4999
7	224	198	303	2825	1322	406	2678	4406
8	781	2048	873	3746	2011	578	4476	7171
9	567	2306	752	3942	1631	316	4723	6679
10	584	2308	749	3954	1945	476	6205	8793
Correlation between manual line–intersect and improved U-net segmentation using root length								
			Pearson’s r		r^2^			*p*-value
Total root length			0.715		0.512			0.020
First-order root			0.270		0.073			0.450
Second-order root			0.106		0.011			0.771
Third-order root			0.880		0.773			<0.001

## Data Availability

The original contributions presented in this study are included in the article/Supplementary Materials. Further inquiries can be directed to the corresponding author.

## Supplementary Materials

The following supporting information can be downloaded at: https://www.mdpi.com/article/10.3390/s25164956/s1, Figure S1: (a) Location of the study area; (b) the photos of rhizotrons installation and image sampling in larch plantation [2]; Table S1: Root window tuber ID, sampling time, and image pixel resolution; Table S2: Pytorch source code for spatial attention, channel attention, and CBAM; Table S3: PyTorch Implementation of Convolutional Block Attention Module (CBAM) and Its Submodules.

## References

[1] Joslin J., Wolfe M., Hanson P. (2000). Effects of Altered Water Regimes on Forest Root Systems. New Phytol..

[2] Huo C., Gu J., Yu L., Wang P., Cheng W. (2022). Temporal Dynamics of Fine Root Production, Mortality and Turnover Deviate across Branch Orders in a Larch Stand. Oecologia.

[3] Krasowski M.J., Lavigne M.B., Olesinski J., Bernier P.Y. (2010). Advantages of Long-Term Measurement of Fine Root Demographics with a Minirhizotron Two Balsam Fir Sites. Can. J. For. Res..

[4] Johnson M.G., Tingey D.T., Phillips D.L., Storm M.J. (2001). Advancing Fine Root Research with Minirhizotrons. Environ. Exp. Bot..

[5] Lei L., Yang Q., Yang L., Shen T., Wang R., Fu C. (2024). Deep Learning Implementation of Image Segmentation in Agricultural Applications: A Comprehensive Review. Artif. Intell. Rev..

[6] Lu W., Wang X., Jia W. (2022). Root Hair Image Processing Based on Deep Learning and Prior Knowledge. Comput. Electron. Agric..

[7] Zeng G., Birchfield S.T., Wells C.E. (2008). Automatic Discrimination of Fine Roots in Minirhizotron Images. New Phytol..

[8] Ronneberger O., Fischer P., Brox T. (2015). U-Net: Convolutional Networks for Biomedical Image Segmentation. Medical Image Computing and Computer-Assisted Intervention—MICCAI 2015.

[9] Gaggion N., Ariel F., Daric V., Lambert É., Legendre S., Roulé T., Camoirano A., Milone D.H., Crespi M., Blein T. (2021). ChronoRoot: High-throughput phenotyping by deep segmentation networks reveals novel temporal parameters of plant root system architecture. Gigascience.

[10] Smith A.G., Petersen J., Selvan R., Rasmussen C.R. (2020). Segmentation of Roots in Soil with U-Net. Plant Methods.

[11] Xu X., Qiu J., Zhang W., Zhou Z., Kang Y. (2022). Soybean Seedling Root Segmentation Using Improved U-Net Network. Sensors.

[12] Wang T., Rostamza M., Song Z., Wang L., McNickle G., Iyer-Pascuzzi A.S., Qiu Z., Jin J. (2019). SegRoot: A High Throughput Segmentation Method for Root Image Analysis. Comput. Electron. Agric..

[13] Tang H., Cheng X., Yu Q., Zhang J., Wang N., Liu L. (2024). Improved Transformer for Time Series Senescence Root Recognition. Plant Phenomics.

[14] Weihs B.J., Heuschele D.-J., Tang Z., York L.M., Zhang Z., Xu Z. (2024). The State of the Art in Root System Architecture Image Analysis Using Artificial Intelligence: A Review. Plant Phenomics.

[15] Yu Q., Wang J., Tang H., Zhang J., Zhang W., Liu L., Wang N. (2023). Application of Improved UNet and EnglightenGAN for Segmentation and Reconstruction of In Situ Roots. Plant Phenomics.

[16] Smith A.G., Han E., Petersen J., Olsen N.A.F., Giese C., Athmann M., Dresboll D.B., Thorup-Kristensen K. (2022). RootPainter: Deep learning segmentation of biological images with corrective annotation. New Phytol..

[17] Dadi M., Lumini A., Franco A. (2025). RootEx: An Automated Method for Barley Root System Extraction and Evaluation. Comput. Electron. Agric..

[18] Woo D., Ghimire A., Jeong S., Kim Y. (2023). Soybean Root Image Dataset and Its Deep Learning Application for Nodule Segmentation. Comput. Electron. Agric..

[19] Xu W., Yu G., Cui Y., Gloaguen R., Zare A., Bonnette J., Reyes-Cabrera J., Rajurkar A., Rowland D., Matamala R. (2022). PRMI: A Dataset of Minirhizotron Images for Diverse Plant Root Study. arXiv.

[20] Huo C., Cheng W. (2019). Improved Root Turnover Assessment Using Field Scanning Rhizotrons with Branch Order Analysis. Ecosphere.

[21] Zhao J.L., Wang J., Qian H.M., Zhan Y.Y., Lei Y. (2022). Extraction of Winter-Wheat Planting Areas Using a Combination of U-Net and CBAM. Agronomy.

[22] Zhang Z.X., Liu Q.J., Wang Y.H. (2018). Road Extraction by Deep Residual U-Net. IEEE Geosci. Remote Sens. Lett..

[23] Woo S., Park J., Lee J.-Y., Kweon I.S. (2018). CBAM: Convolutional Block Attention Module. Lecture Notes in Computer Science.

[24] Kervadec H., Bouchtiba J., Desrosiers C., Granger E., Dolz J., Ben Ayed I. Boundary loss for highly unbalanced segmentation. Proceedings of the 2nd International Conference on Medical Imaging with Deep Learning (MIDL).

[25] Abraham N., Khan N.M., IEEE A Novel Focal Tversky Loss Function with Improved Attention U-Net For Lesion Segmentation. Proceedings of the 16th IEEE International Symposium on Biomedical Imaging (ISBI).

[26] Yu C., Li D., Song M., Yu H., Chang Chein I. (2024). Edge-perception Enhanced Segmentation Method for High-Resolution Remote Sensing Image. Natl. Remote Sens. Bull..

[27] Thesma V., Mohammadpour Velni J. (2023). Plant Root Phenotyping Using Deep Conditional GANs and Binary Semantic Segmentation. Sensors.

[28] Bodner G., Alsalem M., Nakhforoosh A., Arnold T., Leitner D. (2017). RGB and Spectral Root Imaging for Plant Phenotyping and Physiological Research: Experimental Setup and Imaging Protocols. J. Vis. Exp..

[29] Mumuni A., Mumuni F. (2025). Automated Data Processing and Feature Engineering for Deep Learning and Big Data Applications: A Survey. J. Inf. Intell..

[30] Zhang Z., Qiu X., Guo G., Zhu X., Shi J., Zhang N., Ding S., Tang N., Qu Y., Sun Z. (2025). An Automated Root Phenotype Platform Enables Nondestructive High-Throughput Root System Architecture Dissection in Wheat. Plant Physiol..

[31] Zhao B., Chen C., Xiao X., Xia S. (2022). Towards a category-extended object detector with limited data. Pattern Recognit..

[32] Chen L.C., Zhu Y., Papandreou G., Schroff F., Adam H. Encoder-Decoder with Atrous Separable Convolution for Semantic Image Segmentation. Proceedings of the 15th European Conference on Computer Vision (ECCV).

[33] Badrinarayanan V., Kendall A., Cipolla R. (2017). SegNet: A Deep Convolutional Encoder-Decoder Architecture for Image Segmentation. IEEE Trans. Pattern Anal. Mach. Intell..

[34] Zhu S.L., Zhang W.J., Yang T.L., Wu F., Jiang Y.H., Yang G.S., Zain M., Zhao Y.Y., Yao Z.S., Liu T. (2024). Combining 2D Image and Point Cloud Deep Learning to Predict Wheat above Ground Biomass. Precis. Agric..

[35] Luo H., Liu X., Wei X., Wu J., Yu Y., Lu W. (2024). Extracting and Predicting Tomato Root Length Phenotype Using Thz Imaging and Ensemble Learning. Trans. Chin. Soc. Agric. Eng..

[36] Yu Q.S., Zhang M., Wang L.L., Liu X.Y., Zhu L.X., Liu L.T., Wang N. (2025). Research on Fine-Grained Phenotypic Analysis of Temporal Root Systems—Improved YoloV8seg Applied for Fine-Grained Analysis of In Situ Root Temporal Phenotypes. Adv. Sci..

