# Supplementary material for: Improved U-Shaped Convolutional Neural Network with Convolutional Block Attention Module and Feature Fusion for Automated Segmentation of Fine Roots in Field Rhizotron Imagery
Source: Sensors (Basel). 2025 Aug 11;25(16):4956. doi: 10.3390/s25164956 (PMC12389817; doi:10.3390/s25164956)
Supplement: Supplementary file 1 [file sensors-25-04956-s001.zip › sensors-3765903-supplementary.pdf]

Supplementary Materials

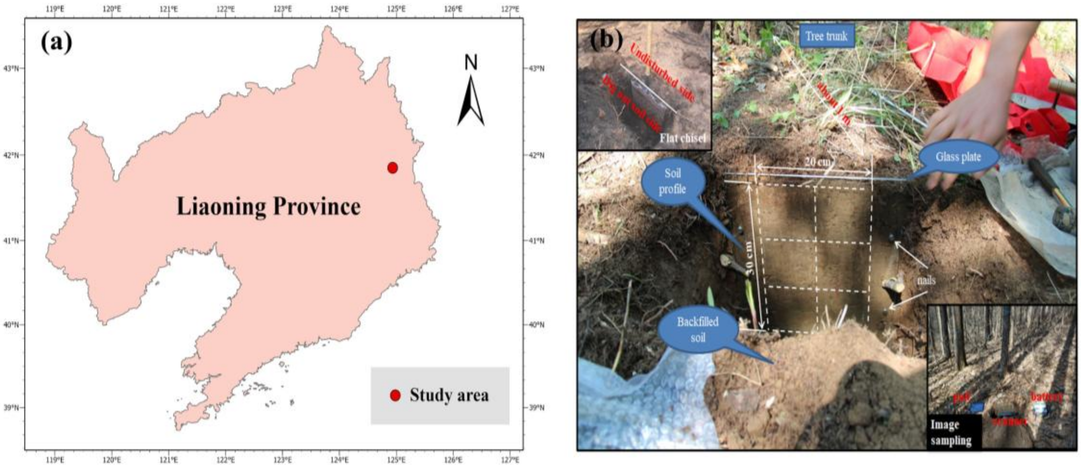

Figure S1. (a) Location of the study area; (b) the photos of rhizotrons installation and image sampling in larch plantation [2].

Table S1. Root window tuber ID, sampling time, and image pixel resolution.

No.	Tube ID	Sampling day (year-month-day)	Pixel (width × height)	Total root length (mm, manually by RootFly)	Total root length (px, calculated by model)
1	Tube 6	2012-7-16	5100 × 7019	225	592
2	Tube 6	2012-8-16	6800 × 9359	1013	1711
3	Tube 6	2012-9-21	6800 × 9359	1967	1410
4	Tube 6	2012-11-10	6800 × 9359	2384	3333
5	Tube 6	2013-5-5	10200 × 14039	2234	2209
6	Tube 6	2013-6-9	10200 × 14039	2779	5519
7	Tube 6	2013-6-29	10200 × 14039	1634	5155
8	Tube 6	2013-7-18	10200 × 14039	1453	6050
9	Tube 6	2013-8-11	10200 × 14039	1438	4591
10	Tube 6	2013-9-6	10200 × 14039	1313	4666
11	Tube 6	2013-10-31	10200 × 14039	1400	6534
12	Tube 6	2014-5-30	10200 × 14039	1356	2536
13	Tube 6	2014-7-13	5100 × 7019	1305	1910
14	Tube 6	2014-9-28	5100 × 7019	1927	2217
15	Tube 6	2014-11-22	5100 × 7019	1922	3261
16	Tube 6	2015-5-16	5100 × 7019	1789	1135
17	Tube 6	2015-6-24	5100 × 7019	1641	2827
18	Tube 6	2015-8-12	5100 × 7019	1006	1487
19	Tube 6	2015-9-18	5100 × 7019	1249	1369
20	Tube 6	2015-11-15	5100 × 7019	1641	2319
21	Tube 6	2016-5-27	5100 × 7019	1606	2114
22	Tube 6	2016-8-7	5100 × 7019	1606	1700

23	Tube 6	2016-11-21	2550 × 3509	1584	3990
24	Tube 6	2017-5-24	2550 × 3509	1437	1731
25	Tube 8	2012-7-16	5100 × 7019	196	602
26	Tube 8	2012-11-10	6800 × 9359	5125	8516
27	Tube 8	2013-5-5	10200 × 14039	4458	6313
28	Tube 8	2013-6-29	5100 × 7019	3618	6446
29	Tube 8	2013-8-11	10200 × 14039	2910	7223
30	Tube 8	2013-10-31	10200 × 14039	2873	6438
31	Tube 8	2014-5-30	10200 × 14039	2912	4861
32	Tube 8	2014-7-13	5100 × 7019	2963	2850
33	Tube 8	2014-8-19	5100 × 7019	3164	3160
34	Tube 8	2014-9-28	5100 × 7019	3523	7990
35	Tube 8	2014-11-22	5100 × 7019	3483	5415
36	Tube 8	2015-5-16	5100 × 7019	3752	5588
37	Tube 8	2015-6-24	5100 × 7019	4010	6165
38	Tube 8	2015-8-12	5100 × 7019	3820	5268
39	Tube 8	2015-9-18	5100 × 7019	3809	860
40	Tube 8	2015-11-15	5100 × 7019	3745	7171
41	Tube 8	2016-5-27	2550 × 3509	3488	4505
42	Tube 8	2016-8-7	5100 × 7019	3941	6679
43	Tube 8	2016-11-21	2550 × 3509	3954	8793
44	Tube 8	2017-5-24	2550 × 3509	3954	5109
45	Tube 12	2012-7-16	5100 × 7019	8.87	71
46	Tube 12	2012-8-16	6800 × 9359	118	586
47	Tube 12	2012-9-21	6800 × 9359	669	1988
48	Tube 12	2012-11-10	6800 × 9359	823	1871
49	Tube 12	2013-5-5	10200 × 14039	657	178
50	Tube 12	2013-6-9	10200 × 14039	1072	2509
51	Tube 12	2013-6-29	10200 × 14039	1211	3347
52	Tube 12	2013-7-18	10200 × 14039	1129	3200
53	Tube 12	2013-8-11	10200 × 14039	961	3238
54	Tube 12	2013-9-6	10200 × 14039	1061	2255
55	Tube 12	2013-9-27	10200 × 14039	1381	3706
56	Tube 12	2013-10-31	10200 × 14039	1438	3742
57	Tube 12	2014-5-30	10200 × 14039	2156	5724
58	Tube 12	2014-7-13	5100 × 7019	2298	5307
59	Tube 12	2014-8-19	5100 × 7019	2148	1681
60	Tube 12	2014-9-28	5100 × 7019	1481	1602
61	Tube 12	2014-11-22	5100 × 7019	1442	1632
62	Tube 12	2015-5-16	5100 × 7019	1474	1914
63	Tube 12	2015-6-24	5100 × 7019	1601	3333
64	Tube 12	2015-8-12	5100 × 7019	1563	1444
65	Tube 12	2015-9-18	5100 × 7019	1278	879
66	Tube 12	2015-11-15	5100 × 7019	1278	2920

67	Tube 12	2016-5-27	5100 × 7019	1290	821
68	Tube 12	2016-8-7	5100 × 7019	1290	1404
69	Tube 12	2016-11-21	2550 × 3509	1171	1079
70	Tube 12	2017-5-24	2550 × 3509	1094	1426
71	Tube 13	2012-7-16	5100 × 7019	235	633
72	Tube 13	2012-8-16	6800 × 9359	381	1115
73	Tube 13	2012-9-21	6800 × 9359	1011	2157
74	Tube 13	2012-11-10	6800 × 9359	1242	4505
75	Tube 13	2013-5-5	10200 × 14039	981	1928
76	Tube 13	2013-6-9	10200 × 14039	854	2904
77	Tube 13	2013-6-29	10200 × 14039	787	4489
78	Tube 13	2014-8-19	5100 × 7019	655	6201
79	Tube 13	2014-9-28	5100 × 7019	864	4999
80	Tube 13	2014-11-22	5100 × 7019	864	5261
81	Tube 13	2015-5-16	5100 × 7019	722	2261
82	Tube 13	2015-8-12	5100 × 7019	675	4406

Table S2. Pytorch source code for Spatial Attention, Channel Attention and CBAM.

Spatial Attention:

```
class SpatialAttention(nn.Module):
    def __init__(self, kernel_size=7):
        super().__init__()
        self.conv = nn.Conv2d(2, 1, kernel_size, padding=kernel_size
// 2)
        self.sigmoid = nn.Sigmoid()

    def forward(self, x):
        avg_out = torch.mean(x, dim=1, keepdim=True)
        max_out, _ = torch.max(x, dim=1, keepdim=True)
        x_cat = torch.cat([avg_out, max_out], dim=1)
        return self.sigmoid(self.conv(x_cat))
```

Channel Attention:

```
class ChannelAttention(nn.Module):
    def __init__(self, in_planes, ratio=16):
        super().__init__()
        self.avg_pool = nn.AdaptiveAvgPool2d(1)
        self.max_pool = nn.AdaptiveMaxPool2d(1)
        self.fc = nn.Sequential(
            nn.Conv2d(in_planes, in_planes // ratio, 1),
            nn.ReLU(),
            nn.Conv2d(in_planes // ratio, in_planes, 1)
        )
        self.sigmoid = nn.Sigmoid()

    def forward(self, x):
        avg_out = self.fc(self.avg_pool(x))
        max_out = self.fc(self.max_pool(x))
        return self.sigmoid(avg_out + max_out)
```

```
CBAM:

class CBAM(nn.Module):
    def __init__(self, in_planes, ratio=16):
        super().__init__()
        self.ca = ChannelAttention(in_planes, ratio)
        self.sa = SpatialAttention()

    def forward(self, x):
        x = x * self.ca(x)
        x = x * self.sa(x)
        return x
```

Table S3. PyTorch Implementation of Convolutional Block Attention Module (CBAM) and Its Submodules.

Module	Code (PyTorch)
Convolutional Block Attention Module	class CBAM(nn.Module):<br>def __init__(self, in_planes, ratio=16):<br>super().__init__()<br>self.ca = ChannelAttention(in_planes, ratio)<br>self.sa = SpatialAttention()

	def forward(self, x):<br>x = x * self.ca(x)<br>x = x * self.sa(x)<br>return x
Channel Attention	class ChannelAttention(nn.Module):<br>def __init__(self, in_planes, ratio=16):<br>super().__init__()<br>self.avg_pool = nn.AdaptiveAvgPool2d(1)<br>self.max_pool = nn.AdaptiveMaxPool2d(1)<br>self.fc = nn.Sequential(<br>nn.Conv2d(in_planes, in_planes // ratio, 1),<br>nn.ReLU(),<br>nn.Conv2d(in_planes // ratio, in_planes, 1)<br>)<br>self.sigmoid = nn.Sigmoid()<br><br>def forward(self, x):<br>avg_out = self.fc(self.avg_pool(x))<br>max_out = self.fc(self.max_pool(x))<br>return self.sigmoid(avg_out + max_out)
Spatial Attention	class SpatialAttention(nn.Module):<br>def __init__(self, kernel_size=7):<br>super().__init__()<br>self.conv = nn.Conv2d(2, 1, kernel_size, padding=kernel_size // 2)<br>self.sigmoid = nn.Sigmoid()<br><br>def forward(self, x):<br>avg_out = torch.mean(x, dim=1, keepdim=True)<br>max_out, _ = torch.max(x, dim=1, keepdim=True)<br>x_cat = torch.cat([avg_out, max_out], dim=1)<br>return self.sigmoid(self.conv(x_cat))
